# Influence of access cavity design on instrumentation efficiency in 3D-printed replicas of mandibular incisors with Vertucci Type III canals

**DOI:** 10.1186/s12903-026-07991-7

**Published:** 2026-02-28

**Authors:** Lu Wang, Panpan Zhang, Wenyuan Zhou, Zhongyang Shan, Chao Liu, Yifen Shen, Nan Geng, Yongchun Gu

**Affiliations:** 1 Department of Dentistry and Central Lab, Ninth People’s Hospital of Suzhou, Soochow University, Suzhou, 215200 China; 2 Department of Otorhinolaryngology, Ninth People’s Hospital of Suzhou, Soochow University, Suzhou, 215200 China; 3 Department of VIP Clinic, State Key Laboratory Cultivation Base of Research, Prevention and Treatment for Oral Diseases, Jiangsu Province Engineering Research Center of Stomatological Translational Medicine, Nanjing Medical University, Affiliated Stomatological Hospital of Nanjing Medical University, 136 Hanzhong Road, Nanjing, 210029 China

**Keywords:** Root canal instrumentation, Mandibular incisor, Double-canaled system, Endodontic access cavity, Micro-computed tomography, Three-dimensional printing

## Abstract

**Background:**

To assess the impact of access cavity design on instrumentation effectiveness in three-dimensional (3D)-printed mandibular incisors with Vertucci Type III canal configuration.

**Methods:**

Two extracted mandibular incisors with Vertucci Type III anatomy, which presented contrasting morphologies (a spacious canal system with isthmuses in Specimen 1 versus a restricted morphology without in Specimen 2), were replicated via 3D printing. This process yielded 48 resin replicas per specimen (total *n* = 96), evenly distributed into six groups (*n* = 8 per group). Each group underwent instrumentation with either WaveOne Gold (WOG) or ProTaper Next (PTN) nickel-titanium (NiTi) systems via one of three access approaches: traditional lingual (TLAC), incisal ridge (IAC), or labial (LaAC). Pre- and post-instrumentation micro-computed tomographic scans were analyzed for iatrogenic errors, canal volume/surface area changes, unprepared areas, and apical diameter. Data were statistically evaluated using three-way analysis of variance (ANOVA) with LSD post hoc test, with the significance level set at *p* < 0.05.

**Results:**

Canal straightening occurred only in the LaAC and IAC groups (9 cases each), whereas apical perforations (22 cases) were exclusive to Specimen 2 replicas. Three-way ANOVA revealed that percentage increases in canal volume and surface area, proportion of unprepared area, and maximum apical diameter following instrumentation were all significantly affected by tooth prototype (all *p* < 0.001), accessing cavity design (all *p* < 0.01), and the instrument used (all *p* < 0.01). In Specimen 1 replicas (spacious canals), PTN with TLAC resulted in a lower proportion of unprepared areas than PTN with LaAC (36.7% ± 8.9% vs. 47.6% ± 7.8%; *p* < 0.05); however, both techniques preparing only one canal. In contrast, PTN with IAC prepared both canals and achieved the most complete preparation (18.8% ± 3.2% unprepared, *p* < 0.05). The use of WOG significantly improved outcomes in the LaAC group, reducing unprepared areas to 13.9% ± 4.8% (*p* < 0.05). Similar trends emerged in Specimen 2 replicas, though the narrower anatomy increased procedural difficulty. WOG consistently outperformed PTN: IAC prepared both canals in all cases (8/8), while LaAC succeeded in 87.5% (7/8). Unprepared surfaces were significantly lower with IAC (8.0% ± 5.5%) and LaAC (9.3% ± 8.2%) than with TLAC (34.2% ± 5.1%; both *p* < 0.01).

**Conclusions:**

IAC demonstrated superior efficacy for instrumentation of mandibular incisors with Vertucci Type III canals compared to TLAC and LaAC. WOG enhanced LaAC performance and surpassed PTN overall. Access design and instrument selection critically influence procedural success.

## Background

Mandibular incisors, recognized as the smallest teeth in the adult dentition, are anatomically characterized by a single root containing a single canal [1, 2]. These roots typically demonstrate a distinct labiolingual flattening, often accompanied by longitudinal depressions along their proximal surfaces [2]. Previous studies have revealed that 11% to 45% of mandibular incisors exhibit complex root canal configurations, with majority presenting as dual canals arranged in a labiolingual orientation [3–13]. Conventional radiographic techniques frequently fail to detect additional canals due to anatomical superimposition, potentially leading to missed canals, incomplete disinfection, and treatment failure [14]. As the current diagnostic gold standard, cone-beam computed tomography (CBCT) provides superior three-dimensional (3D) visualization of root canal systems. More recently, the dynamic navigation system (DNS) has been introduced to further improve clinical precision, enabling accurate access cavity preparation and root canal orifice localization while minimizing dentin removal [15, 16]. Despite these advancements, clinical management remains technically challenging. Even in cases with a confirmed single canal, the high prevalence of oval canal configurations poses significant challenges for complete debridement [2, 17–20]. Furthermore, although modern endodontic instrumentation systems have made technological progress, they often exhibit limited efficacy in fully eliminating biofilm from these complex anatomical spaces.

Access cavity design critically impacts subsequent instrumentation effectiveness, disinfection, and fracture resistance [16, 21]. For mandibular incisors, the traditional lingual access cavity employs a conservative lingual approach featuring an ovoid outline centered on the cingulum. While this design effectively preserves labial dentin and maintains the incisal edge integrity, its minimal mesiodistal extension, though typically adequate for primary canal location, may restrict visualization of additional canals [22]. Incisally shifted access approaches may provide superior visualization and instrumentation, particularly in incisors with oval canal configurations, while preserving critical pericervical dentin [23–26]. Logani et al. [27] demonstrated that a labial access cavity for mandibular incisors provides more consistent straight-line access to root canals while achieving greater conservation of tooth structure. However, this approach compromises facial esthetics, and its clinical efficacy for ensuring complete debridement in anatomically complex cases requires further investigation.

Minimally invasive (MI) or conservative endodontic cavity (CEC) aim to preserve crucial tooth structures, particularly the pericervical dentin, a key component in stress distribution [28]. However, such constrained approach may compromise fundamental procedural goals, including straight-line access, thorough debridement, and optimal obturation. A previous study [22] on mandibular incisors found no significant difference in fracture resistance between MI and traditional access, suggesting limited mechanical benefits of MI. MI access was linked to inferior quality of root canal obturation, underscoring a critical trade-off between structural preservation and sealing effectiveness. More recently, Liu et al. [16] reported that both CEC and modified CEC prepared with DNS could preserve more dentin and exhibited higher fracture resistance than freehand-prepared traditional access in mandibular first premolars with Vertucci type V canals. In severely curved type V canal, modified CEC achieved comparable outcomes in instrumentation and obturation to traditional access. This controversy indicates that the choice between MI and traditional approaches depends mainly on specific tooth anatomy [16].

The introduction of nickel-titanium (NiTi) systems, renowned for their superior flexibility and shape memory, has revolutionized root canal preparation. ProTaper Next (PTN) is among the most established rotary systems and is frequently employed as a reference in comparative research. Its asymmetric rectangular cross-section induces a characteristic off-center rotation that improves canal tracking and minimizing transportation. Furthermore, its efficacy and preservation of canal anatomy have been specifically evaluated in several studies focusing on mandibular incisors [29–31]. Single-file alternatives like WaveOne Gold (WOG) incorporate: (1) an off-center parallelogram cross-section, (2) four size options (20/7% to 45/5%), and (3) gold heat treatment for enhanced flexibility and fatigue resistance [32–36]. WOG operates via a 170° counterclockwise cutting/50° clockwise release reciprocating motion [33, 36, 37]. Despite the notable efficiency advantages of WOG, PTN remains more extensively documented in comparative studies; both systems, however, demonstrate reliable efficacy in managing curved canals.

3D printing enables production of anatomically precise tooth replicas, thus overcoming the limitations associated with extracted teeth, such as infection risks, ethical concerns, and morphological variability [32, 38]. Radiopaque 3D-printed replicas allow for noninvasive micro-computed tomography (micro-CT) evaluation at micron-level resolution [32]. In this study, micro-CT was used to evaluate the influence of access cavity design (IAC, TLAC, or LaAC) and NiTi system (WOG or PTN) on instrumentation outcomes in 3D-printed mandibular incisors with Vertucci Type III canals. The null hypothesis was that neither access design nor instrumentation system would significantly affect canal preparation outcomes.

## Materials and methods

Approval for use of extracted teeth was obtained from the Institutional Ethics Committee for human research (Approval Number: KY2022-089-01), and informed consent was obtained from all subjects.

### Specimen selection

**Fig. 1:**
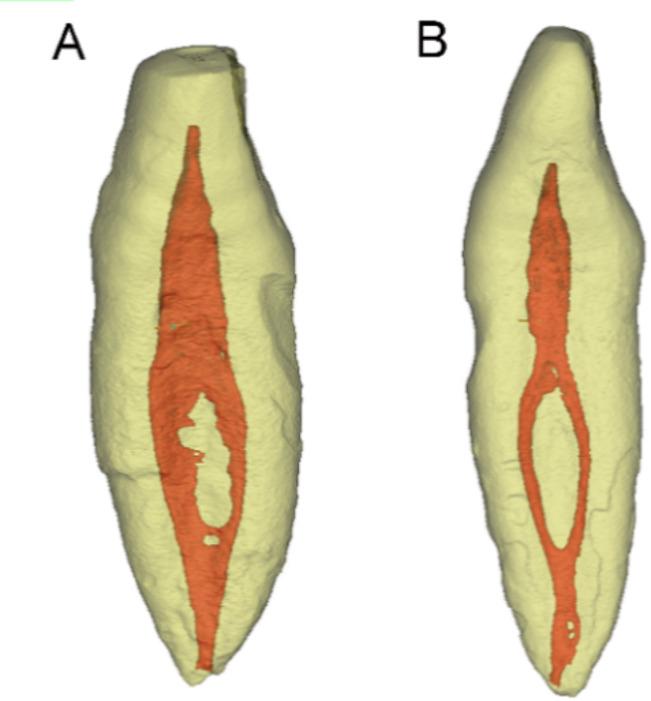
Three-dimensional micro-CT reconstructions of two representative double-canaled mandibular incisors selected as prototypes for 3D printing (proximal view). **A** Specimen 1 demonstrates a spacious root canal system with detectable canal isthmuses; (**B**) Specimen 2 exhibits a constricted canal morphology with no observable isthmus formation

Micro-CT image datasets from 106 extracted permanent mandibular incisors were obtained from a previous investigation [2]. Scanning was performed using a SkyScan1174 system (Bruker-micro-CT, Kontich, Belgium). The acquisition parameters were set as follows: 50 kV voltage, 800 µA current, and a 9 μm voxel size, with a 1 mm aluminum filter applied. The imaging process employed a 180° rotation, a step size of 0.7°, and an averaging of 1 frame per projection. Analysis of root canal configurations revealed a 22.6% prevalence (24/106) of double canal systems, with the majority (21/24) demonstrating buccal and lingual canal convergence prior to the apex [2]. From this sample collection, two representative specimens exhibiting Vertucci Type III canal configuration (1-2-1) (Fig. 1) were selected as prototypes for 3D printing. Specimen 1 demonstrated a spacious root canal system with detectable canal isthmuses. In contrast, Specimen 2 exhibited a constricted canal morphology with no observable isthmus formation. Both incisors showed similar crown-root dimensions, measuring 19.5 mm (Specimen 1) and 20.0 mm (Specimen 2) in length. Schneider’s method [39] analysis revealed severe canal curvature (> 20°) affecting both buccal and lingual canals in each specimen.

### Micro-CT imaging of the prototype teeth

The two selected prototype teeth were rescanned using the same micro-CT system (SkyScan 1174). The scan was optimized for model fidelity, achieving a high isotropic voxel resolution of 21.6 μm under parameters of 50 kV voltage, 800 µA current, 180° rotation, and a 0.7° incremental step size with a 1 mm aluminum filter. Image reconstruction was performed using Mimics 21 software (Materialise, Leuven, Belgium).

### 3D printing of mandibular incisors with double canals

The tooth replicas were fabricated using an established 3D printing protocol [32]. To ensure effective removal of uncured resin following polymerization, dedicated drainage channels with diameters ranging from 0.25 to 0.50 mm were digitally integrated into the design of each replica. In Mimics software, cylindrical structures, defined by specific diameter and length parameters, were created to connect the pulp chamber to the labial/buccal surface and the incisal edge. These cylinders were then subtracted from the replica models using Boolean subtraction operations, resulting in hollow conduits that functioned as drainage pathways. Digital models were then exported in standard triangle language (STL) format and manufactured using a high-precision resin-based 3D printer (Saturn 2, ELEGOO, China) with spatial resolutions of 28.5 μm (XY plane) and 20 μm (Z-axis), employing MOLEGRID™ Ultradetail photopolymer resin (KEXCELLED, China). The resin-evacuation channels enabled thorough ethanol irrigation to remove uncured resin residues after photopolymerization, which is a critical step for preserving accurate root canal anatomy. This protocol yielded 96 identical tooth replicas (*n* = 48 per prototype specimen).

### Access cavities preparation

**Fig. 2:**
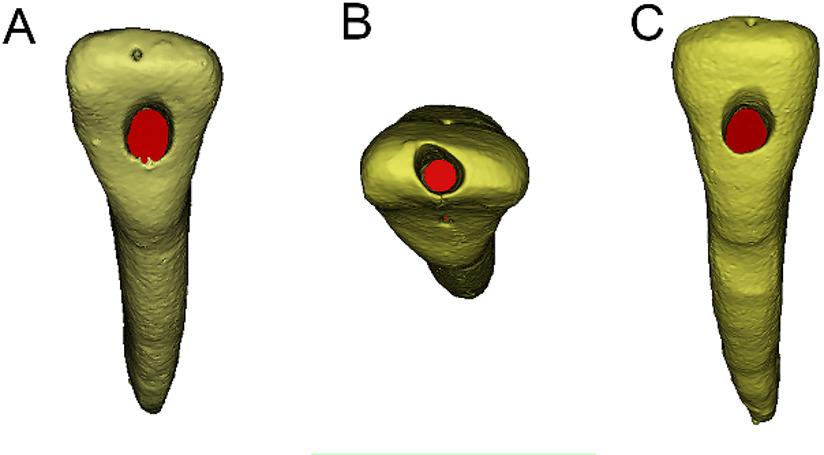
Three access cavity designs for mandibular incisors (representative micro-CT 3D images of Specimen 2 replicas). **A** Traditional lingual access cavity (lingual view); (**B**) incisal access cavity (incisal view); (**C**) labial access cavity (labial view)

Forty-eight replicas from each specimen were randomly assigned to six experimental groups (*n* = 8 per group): TLAC/PTU, TLAC/WOG, IAC/PTU, IAC/WOG, LaAC/PTU, and LaAC/WOG. Each group was subjected to a distinct access cavity preparation approach (lingual, incisal ridge, or labial) (Fig. 2), followed by root canal instrumentation using either the WOG or PTN system. All instrumentation procedures were performed by a single operator (L.W.), a general dentist with two years of clinical experience, to eliminate inter-operator variability. The operator was informed that all replicas featured a Vertucci Type III (1-2-1) canal configuration to ensure standardized procedural planning and consistency across specimens. However, the operator was not shown the detailed preoperative micro-CT images of the two prototype teeth and remained unaware of specific anatomical variations (e.g., presence or absence of isthmuses, degree of canal constriction). Prior to the study, the operator underwent specialized hands-on training for each NiTi system, utilizing additional identical 3D-printed replicas of the study prototypes, under the direct supervision of an experienced endodontist (Y.G.) with over 27 years of clinical practice. Training continued until consistent and reproducible proficiency was demonstrated, ensuring full adherence to the manufacturers’ guidelines. This calibration approach allowed the operator to achieve consistent performance while becoming familiar with the general anatomical features of the Vertucci Type III configuration, mirroring contemporary clinical practice where preoperative CBCT typically reveals the presence and approximate layout of complex canal systems. Access cavities were prepared using round-end tapered diamond burs (F102R, Shofu, Kyoto, Japan) under continuous water cooling at high-speed rotation.

- Traditional lingual access cavity (TLAC) [22, 25]: Following initial penetration 1 mm incisal to the cingulum on the lingual surface, the access cavity was developed along cervico-incisal and mesiodistal axes to completely unroof the pulp chamber. Strategic removal of lingual pericervical dentin subsequently facilitated direct root canal entry.
- Incisal access cavity (IAC): The access cavity was prepared at the incisal edge while conserving the cingulum and coronal dentin. Initial penetration was performed using a tapered diamond bur aligned parallel to the tooth’s long axis to ensure proper orientation. The final cavity design maintained an ovoid contour centered at the incisal edge, with a maximum width of 2.0 mm to optimize both structural preservation and endodontic access.
- Labial access cavity (LaAC) [27]: LaAC was prepared with the initial entry point positioned slightly occlusal to the mid-labial surface of the tooth. A tapered diamond bur was oriented perpendicular to the long axis to penetrate the artificial crown structure. Following initial access, circumferential refinement completely removed the pulp chamber roof in both cervicoincisal and mesiodistal dimensions, ensuring complete orifice exposure while preserving pericervical dentin.

Access cavity preparations were performed using a standardized conservative protocol across all groups to preserve pericervical dentin and maintain experimental comparability. This involved complete unroofing of the pulp chamber and adequate initial orifice visibility/access —adequate for orifice location using DG-16 explorers and glide path establishment with #8 to #15 K-files (Dentsply Sirona, Ballaigues, Switzerland)—without aggressive reduction of lingual or buccal shoulders or overhangs. No orifice flaring instruments (e.g., Gates-Glidden drills or ultrasonic tips) were employed, as such aggressive techniques in resin replicas are prone to causing irreversible deformation, ledge formation, perforation, or obstruction by melted debris, which would necessitate specimen replacement and undermine the essential standardization required for valid inter-group comparisons. This conservative approach reflects current emphasis on dentin preservation [28] and created equivalent coronal constraints for fair comparison of access designs.

### Root canal instrumentation by NiTi systems

After access cavity preparation, canal orifices were mapped using DG-16 explorers, with supplementary canal exploration employing pre-curved #10 K-files. Following confirmation of apical patency by passively inserting a #8 and a #10 K-file, glide path preparation was established with a #15 K-file at the established working length (WL), located 1 mm short of the apical foramen. Notably, no orifice openers or magnification aids were employed throughout the procedures. Instrumentation was performed using an X-Smart Plus endodontic motor (Dentsply Maillefer, Switzerland) under strict single-use protocols. The debris on the files was cleaned with wet gauze, and the canal was irrigated with 2 mL of distilled water each time.

- Group PTN: The sequence of instruments was: X1 (17/0.04), and X2 (25/0.06). Each instrument was passively introduced into the root canal at a 300 rpm rotation rate and 2.5 N/cm torque. The instruments were used in a slow in-and-out motion of approximately 3 mm in amplitude in the apical direction, using a gentle brushing motion against the canal wall. The procedure was repeated twice until the WL was reached.
- Group WOG: The WOG Primary instrument (25/0.07) was used in a reciprocating motion (using at 350 rpm with the “WAVEONE ALL” program). Slight apical pressure was applied during sample preparation. After short consecutive movements of penetration and removal, the instrument was removed from the canal and cleaned using a piece of sterile gauze. These procedures were repeated until the file reached the original WL.

### Micro-CT analysis of tooth replicas

The resin replicas were scanned via micro-CT before and after root canal preparation according to identical scanning parameters (voxel size 21.6 μm, 50 kV, 800 µA, 180° rotation, 0.7° step size). Pre- and post-instrumentation images were geometrically coregistered using the DataViewer software v1.5.2 (Bruker-microCT, Kontich, Belgium), and then the 3D models of the teeth and root canal systems were reconstructed using Mimics software. The region of interest extended from the cementoenamel junction (CEJ) to the root apex, with the following geometric parameters related to root canal instrumentation being analyzed:

Canal volume and surface area: The volumetric and surface area measurements of the root canal system were quantified both before and after instrumentation. The percentage changes in volume and surface area (% ∆) were calculated using the formula: % ∆ = ([A-B] / B) × 100, where A represents the post-instrumentation data and B denotes the pre-instrumentation data.

Unprepared surface areas: The evaluation of the unprepared areas within the root canal was conducted using a Boolean operation in the Mimics software, adhering to the methodology outlined in previous studies [40, 41]. Surface voxels that remained unchanged in position from preoperative to postoperative assessments represented the uninstrumented regions of the canal wall. This parameter was quantified as the percentage of static surface voxels relative to the total number of pre-instrumentation surface voxels, providing a precise measure of the canal areas that remained untouched by the instrumentation process.

The maximum (*D*) and minimum diameters (*d*) of the apical foramen at the WL were measured both before and after instrumentation, and the *D*/*d* ratio was calculated to quantify the deviation from roundness.

The following iatrogenic errors were recorded based on pre- and post-instrumentation micro-CT analyses: (a) Apical perforation: Visualization of an artificial communication between the root canal and the external root surface within the apical 3 mm. (b) Canal straightening: At the proximal view, a post-instrumentation root canal curvature of less than 10°, as measured by Schneider’s method [39]. This criterion was established due to the severe initial curvatures (> 20°) at the canal divergence and convergence sites in the Type III (1-2-1) specimens. (3) Instrument separation: The presence of a fractured instrument segment within the root canal system.

### Statistical analysis

Sample size calculation was estimated a priori using G*Power software (version 3.1.9.7; Heinrich-Heine Universität Düsseldorf, Düsseldorf, Germany). Based on preliminary pilot data, an effect size (*f*) of 0.40 was assumed for the primary outcome. With an alpha level of 0.05 and a desired statistical power of 0.8, the calculation indicated a minimum total sample size of 64 specimens. Given this, and considering the highly standardized nature of the 3D-printed replicas, which significantly reduces inter-specimen variability compared to natural extracted teeth, a sample size of eight replicas per group was adopted for this model, aligning with common sample sizes (typically 6 to 10 per group) reported in comparable ex vivo endodontic studies [16, 25, 26, 29, 32]. Consequently, the study utilized eight replicas per experimental group, resulting in a total sample size of 96, which exceeds the calculated minimum requirement. All statistical analyses were conducted with IBM SPSS Statistics (Version 26.0; IBM Corp., USA). The normality of data was assessed using the Shapiro-Wilk test. Subsequently, for multiple-group comparisons, a three-way analysis of variance (ANOVA) was conducted, followed by the Least Significant Difference (LSD) post hoc test. A significance level of 5% was used.

## Results

**Fig. 3:**
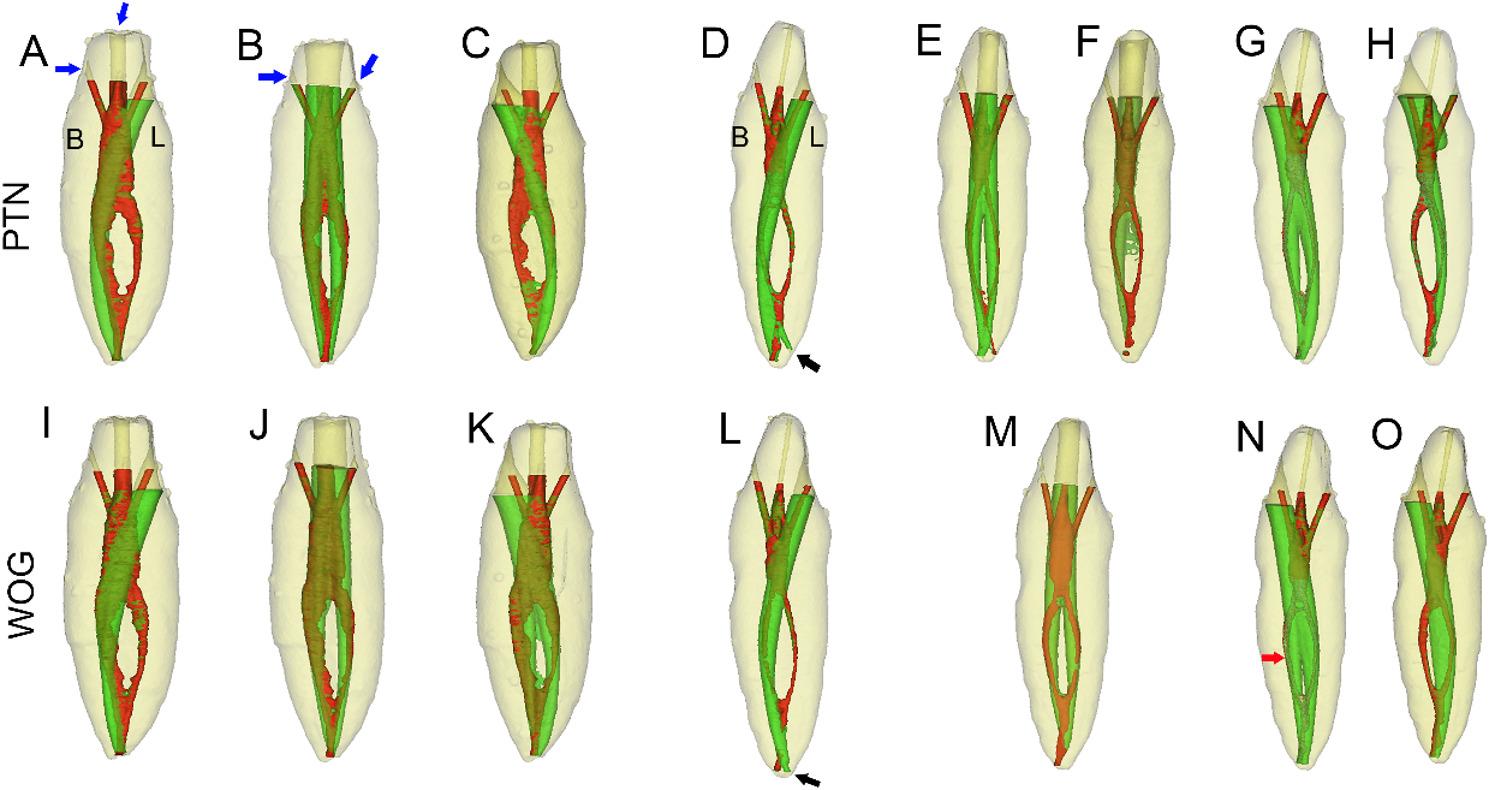
Micro-CT 3D reconstructions and segmentations of double-canaled mandibular incisor replicas prepared with PTN and WOG using three different access cavity designs. **A**-**C** Specimen 1 replicas prepared with PTN using TLAC (**A**), IAC (**B**), and LaAC (**C**). **D**-**H** Specimen 2 replicas prepared with PTN via TLAC (**D**), IAC (**E** and **F**), and LaAC (**G** and **H**) approaches. **I**-**K** Specimen 1 replicas prepared with WOG using TLAC (**I**), IAC (**J**), and LaAC (**K**) approaches. **L**-**O** Specimen 2 replicas prepared with WOG via TLAC (**L**), IAC (**M**), and LaAC (**N** and **O**) approaches. Black arrows indicate apical perforations; red arrow indicates canal straightening. Blue arrows mark drainage channels for ethanol rinsing of uncured resin post-polymerization

The homogeneity of the groups with respect to the initial canal volume, and surface area was confirmed (*p* > 0.05), indicating no significant differences in baseline morphological parameters among the experimental groups. Fig. 3 displays 3D reconstructions and segmentations of double-canaled mandibular incisor replicas prepared with PTN and WOG using three different access cavity designs.

Occurrence of iatrogenic errors during root canal instrumentation, including unprepared canal, instrument separation, apical perforation and canal straightening were presented in Table 1. Instrument separation occurred in four cases. Specifically, three events happened in Specimen 1 replicas (one in the TLAC group using PTN X2, one in the LaAC group using PTN X2, and one in the LaAC group using WOG). The remaining case was in a Specimen 2 replica from the TLAC group with WOG. The affected tooth replicas were subsequently replaced. Canal straightening was exclusively observed in the LaAC and IAC groups (nine cases each), with no occurrences detected in the TLAC group. Apical perforation was exclusively observed in Specimen 2 replicas (22 cases), predominantly associated with TLAC approach (13 cases).

**Table 1:** Occurrence of iatrogenic errors during root canal instrumentation (*n* = 8 per group) *n*

NiTi system	Access cavity	Unprepared buccal canals	Unprepared lingual canals	Instrument separation	Apical perforation	Canal straightening
Specimen 1 replicas						
PTN	TLAC	0	8	1	0	0
	IAC	0	0	0	0	0
	LaAC	8	0	1	0	0
WOG	TLAC	0	8	0	0	0
	IAC	0	0	0	0	4
	LaAC	0	0	1	0	7
Specimen 2 replicas						
PTN	TLAC	0	8	0	6	0
	IAC	0	3	0	3	5
	LaAC	5	0	0	0	0
WOG	TLAC	0	8	1	7	0
	IAC	0	0	0	3	0
	LaAC	1	0	0	3	2

*PTN* ProTaper Next, *WOG *WaveOne Gold, *TLAC* denotes traditional lingual access cavity, *IAC* denotes incisal access cavity, *LaAC* denotes labial access cavity

In Specimen 1 replicas featuring spacious canal dimensions and detectable isthmus, PTN instrumentation with TLAC approach consistently enabled preparation of all buccal canals; however, this approach uniformly failed to provide access to lingual canals. In contrast, the IAC technique reliably achieved access to both canals in all cases. When employing the LaAC approach, PTN instrumentation successfully prepared all lingual canals but failed to instrument any buccal canals; however, switching to WOG instrumentation significantly enhanced the preparation efficacy, achieving complete instrumentation of both canals in all specimens (8/8). Notably, this improved performance was accompanied by a high incidence of canal straightening (87.5%, 7/8). A comparable trend was observed in Specimen 2 replicas, though the increased anatomical complexity of the narrowed pulp space elevated operational challenges. The TLAC approach with PTN instrumentation demonstrated complete preparation of all buccal canals (8/8), while failing to instrument any lingual canals (0/8), accompanied by a concerning 75.0% (6/8) apical perforation rate. The IAC technique achieved complete dual-canal instrumentation in 62.5% of cases (5/8), with the remaining 37.5% (3/8) limited to buccal canal preparation due to obturation or ledging in the lingual canal. With the LaAC approach, PTN instrumentation achieved complete preparation of both canals in 3 specimens but failed to access buccal canals in the remaining 5 cases. Transitioning to WOG instrumentation markedly improved outcomes: the IAC technique achieved 100% (8/8) complete preparation, while the LaAC approach succeeded in 87.5% of cases (7/8), with 1 specimen showing lingual-only instrumentation. Notably, both IAC and LaAC approaches demonstrated substantially lower apical perforation rates (37.5%, 3/8 each) compared to the TLAC approach (87.5%, 7/8). Quantitative analysis revealed distinct preparation efficacy among the techniques (Tables 2, 3, 4 and 5).A three-way ANOVA indicated that the percentage increases in canal volume and surface area, the proportion of unprepared surface area, and the apical diameters after instrumentation were all significantly affected by tooth prototype, accessing cavity design, and the instrument used (all *p* < 0.01). Post hoc comparisons revealed that in Specimen 1 replicas, PTN instrumentation with IAC approach achieved the most pronounced increase in canal volume and surface area, and the smallest unprepared areas (18.8%); in contrast, the TLAC and LaAC approaches produced significantly less canal enlargement, with LaAC producing the largest unprepared areas (47.6%) (all *p* < 0.05). The use of WOG instrumentation significantly reduced unprepared areas for both the LaAC and IAC approaches compared to TLAC. The unprepared area for LaAC was reduced from 34.4% (with TLAC) to 13.9%, and for IAC, it was reduced to 11.8% (both *p* < 0.05). A similar trend was observed in Specimen 2 replicas with PTN instrumentation, though statistical significance was only achieved for unprepared areas (IAC:11.6%, LaAC:29.2%, TLAC:30.8%; *p* < 0.05). Notably, WOG instrumentation significantly enhanced preparation efficacy, particularly in buccal canals via the LaAC technique, producing significantly greater volumetric and surface area expansion than the TLAC approach (all *p* < 0.05). Furthermore, with WOG instrumentation, the LaAC technique yielded comparable unprepared areas (9.3%) to the IAC technique (8.0%; *p* > 0.05), and both were significantly lower than the 34.2% observed with TLAC (*p* < 0.05).

**Table 2:** Volume, surface area, and unprepared canal surface of double-canal systems in mandibular incisor replicas before and after instrumentation (*n* = 8 per group)

NiTi system	Access cavity	Initial volume (mm^3^)	Final volume (mm^3^)	Volume increase (%)	Initial surface area (mm^2^)	Finalsurface area (mm^2^)	Surface area increase (%)	Unprepared surface area (%)
Specimen 1 replicas								
PTN	TLAC	5.85±0.42^a^	10.22±1.21^b^	75.8±26.3^a^	38.88±1.65^a^	48.40±3.85^b^	24.6±10.2^b^	36.7±8.9^b^
	IAC	5.76±0.45^a^	13.30±1.06^a^	132.6±28.6^a^	38.46±1.25^a^	56.81±2.13^a^	47.8±6.8^a^	18.8±3.2^a^
	LaAC	5.97±0.33^a^	10.72±0.71^b^	80.2±16.8^a^	40.10±1.50^a^	53.04±2.46^c^	32.4±6.1^ab^	47.6±7.8^c^
WOG	TLAC	5.67±0.30^a^	10.38±0.58^b^	83.6±15.1^b^	38.06±1.37^a^	49.71±3.74^b^	30.8±7.7^b^	34.4±8.4^b^
	IAC	5.70±0.45^a^	13.71±1.09^a^	140.9±14.7^a^^b^	38.59±1.09^a^	58.39±3.22^a^	51.3±6.5^a^	11.8±4.4^a^
	LaAC	5.60±0.45^a^	14.76±1.31^a^**	164.4±22.2^a^**	38.04±1.98^a^	58.98±4.09^a^**	55.1±8.3^a^*	13.9±4.8^a^**
Specimen 2 replicas								
PTN	TLAC	2.96±0.12^a^	8.26±1.53^a^	179.8±54.7^a^	25.71±0.91^a^	41.79±3.74^a^	56.8±10.1^b^	30.8±5.7^b^
	IAC	2.95±0.16^a^	11.40±2.87^b^	289.3±107.7^b^	26.17±0.70^a^	48.48±7.53^a^	75.8±22.3^a^	11.6±10.1^a^
	LaAC	3.00±0.14^a^	10.37±3.77^b^	248.1±135.4^b^	26.11±0.93^a^	47.51±9.48^a^	63.8±17.0^a^	29.2±18.5^b^
WOG	TLAC	2.78±0.35^a^	8.66±0.61^b^	214.7±39.3^a^	25.18±1.97^a^	42.61±2.24^b^	69.7±8.7^b^	34.2±5.1^b^
	IAC	2.93±0.28^a^	10.70±1.53^a^	270.2±79.6^a^	26.04±1.66^a^	50.79±4.12^a^	96.4±27.3^a^	8.0±5.5^a^
	LaAC	2.87±0.12^a^	13.01±1.58^c^**	355.8±70.5^b^	26.04±0.52^a^	53.54±4.36^a^	105.9±19.7^a^*	9.3±8.2^a^**

Data were analyzed by three-way ANOVA. Values with the different lowercase superscript letters along the same column are significantly different (*p *< 0.05)

*TLAC* denotes traditional lingual access cavity, *IAC* denotes incisal access cavity, *LaAC* denotes labial access cavity

Statistical significance between the two NiTi systems for the same access cavity design is denoted as *p < 0.05 and **p < 0.01

**Table 3:** Three-way ANOVA results for the association of the independent variables and three dependent variables

	Volume increase (%)		Surface increase (%)		Unprepared area (%)	
	F	*p*	F	*p*	F	*p*
Tooth prototype	127.776	0.000	130.158	0.000	14.869	0.000
Access cavity design	13.591	0.004	15.731	0.000	51.513	0.000
NiTi instrument	8.252	0.005	10.522	0.002	36.800	0.000

**Table 4:** The measurement results of maximum (*D*) and minimum diameters (*d*) of the apical foramen at the working length pre- and post-instrumentation. (*n* = 8 per group)

NiTi system	Access cavity	Pre-instrumentation			Post-instrumentation		
		D (mm)	d (mm)	D/d	D (mm)	d (mm)	D/d
Specimen 1 replicas							
PTN	TLAC	0.366±0.039^a^	0.286±0.048^a^	1.297±0.138^a^	0.503±0.108^b^	0.377±0.022^b^	1.331±0.253^b^
	IAC	0.350±0.052^a^	0.285±0.043^a^	1.238±0.166^a^	0.652±0.107^a^	0.389±0.010^ab^	1.689±0.291^a^
	LaAC	0.350±0.064^a^	0.272±0.040^a^	1.290±0.166^a^	0.628±0.120a	0.424±0.076^a^	1.517±0.296^a^^b^
WOG	TLAC	0.355±0.037^a^	0.287±0.018^a^	1.240±0.191^a^	0.672±0.073^b^**	0.377±0.019^a^	1.790±0.169^b^**
	IAC	0.365±0.027^a^	0.313±0.020^a^	1.203±0.123^a^	0.658±0.070^b^	0.408±0.016^a^	1.608±0.171^b^
	LaAC	0.356±0.049^a^	0.284±0.030^a^	1.273±0.150^a^	0.797±0.219^a^**	0.413±0.050^a^	2.357±0.396^a^**
Specimen 2 replicas							
PTN	TLAC	0.344±0.050^a^	0.215±0.030^a^	1.603±0.091^a^	0.460±0.062^a^	0.321±0.047^b^	1.436±0.064^a^
	IAC	0.359±0.061^a^	0.214±0.039^a^	1.683±0.224^a^	0.545±0.112^a^	0.374±0.055^a^	1.455±0.187^a^
	LaAC	0.368±0.024^a^	0.238±0.022^a^	1.553±0.151^a^	0.538±0.094^a^	0.362±0.022^a^	1.471±0.185^a^
WOG	TLAC	0.325±0.054^a^	0.203±0.028^a^	1.595±0.106^a^	0.679±0.155^a^**	0.349±0.060^b^	2.026±0.533^b^**
	IAC	0.355±0.067^a^	0.213±0.041^a^	1.682±0.197^a^	0.449±0.045^b^	0.270±0.028^c^	1.677±0.196^a^
	LaAC	0.356±0.071^a^	0.239±0.023^a^	1.487±0.246^a^	0.786±0.165^a^**	0.400±0.022^a^	1.888±0.403^ab^**

Data were analyzed by three-way ANOVA. Values with the different lowercase superscript letters along the same column are significantly different (*p *< 0.05)

*TLAC* denotes traditional lingual access cavity, *IAC* denotes incisal access cavity, *LaAC* denotes labial access cavity, *D* denotes maximum diameter, and *d* denotes minimum diameter

Statistical significance between the two NiTi systems for the same access cavity design is denoted as *p < 0.05 and **p < 0.01

**Table 5:** Three-way ANOVA results for the association of the independent variables and apical diameters after instrumentation

	D		d		D/d	
	F	*p*	F	*p*	F	*p*
Tooth prototype	20.815	0.000	38.040	0.000	0.591	0.444
Access cavity design	20.072	0.000	11.042	0.000	5.350	0.007
NiTi instrument	42.197	0.000	0.360	0.550	51.775	0.000

*D* denotes maximum diameter, and *d* denotes minimum diameter

Apical diameter measurements are summarized in Tables 4 and 5. In Specimen 1 replicas, the LaAC/WOG combination produced the largest *D* and the highest *D/d* ratio. In Specimen 2 replicas, LaAC/WOG again yielded the largest *D*, whereas TLAC/WOG resulted in the highest *D/d* ratio.

## Discussion

This study utilized two double-canaled mandibular incisors with distinct anatomical features: one with wide, patent canals and detectable isthmuses, and the other with narrow, constricted canals without isthmus formation. Both specimens exhibited a type 1-2-1 canal configuration, which is the most prevalent form in multi-canaled mandibular incisors [2]. Corresponding resin replicas were fabricated via high-precision 3D printing technology. Preoperative geometric measurements revealed no significant intergroup differences, confirming morphological consistency across specimens. Conventional ex vivo instrumentation studies predominantly rely on extracted human teeth. However, natural teeth exhibit inherent anatomical variability, complicating specimen standardization and balanced group allocation. Moreover, double-canaled mandibular incisors are clinically scarce, making procurement of sufficient intact specimens challenging. Our findings align with prior research [32] demonstrating that 3D-printed replicas enhance experimental consistency, reproducibility, and repeatability. These models provide clinically accurate simulations, establishing a reliable platform for evaluating the impact of access cavity designs on the preparation of mandibular incisors with Vertucci Type III canal configurations.

The design of endodontic access cavities critically influences treatment success by ensuring complete pulp tissue removal, facilitating canal orifice identification, and enabling effective irrigant delivery [22, 42]. In anterior teeth, TLACs are typically prepared in the cingulum region due to its anatomical proximity to the pulp chamber and aesthetic advantages. The preparation is frequently extended into the pericervical dentin to facilitate improved instrument access to apical regions and enhance treatment efficacy [25, 43]. While TLAC preserves labial structure and follows the buccal canal trajectory, it consistently fails to provide adequate access to the lingual canal due to coronal interference, resulting in 30.8%-36.7% of canal surfaces remaining unprepared, including critical isthmus areas that may retain biofilm. Moreover, despite minimizing incisal resin removal, TLAC requires substantial cervical dentin reduction, potentially compromising fracture resistance [44]. Notably, TLAC is associated with a higher incidence of apical perforations (13 cases) compared to IAC (6 cases) and LaAC (3 cases). These findings indicate that clinicians should carefully weigh the aesthetic advantages of TLAC against its mechanical risks (particularly in patients with parafunctional habits or pre-existing cervical tooth structure loss), as well as the risk of missing additional canals. Preoperative CBCT evaluation of remaining dentin thickness and accessory lingual canal morphology facilitates case selection for alternative access designs that optimize both complete debridement and structural preservation. Clinically, this means that for patients where aesthetics is paramount and the risk of lingual canal missed is low (e.g., confirmed single canal via CBCT), TLAC may be considered, but it should be avoided in cases with preoperative evidence of complex anatomy or cervical dentin loss to prevent catastrophic failures.

The access cavity preparations in this study intentionally adopted a conservative approach to pericervical dentin removal, limiting aggressive reduction of lingual shoulders (TLAC) or buccal overhangs (LaAC) while ensuring complete pulp chamber unroofing and adequate initial orifice access. This standardized conservative protocol was applied uniformly across all groups to preserve experimental consistency in resin replicas, where excessive dentin/resin removal risks irreversible ledges, perforations, or deformation that would require specimen replacement. By creating equivalent coronal constraints, this design functioned as a controlled “stress test” isolating differences attributable to access location and angulation rather than variable degrees of shoulder modification. The resulting increased technical difficulty—evident in higher rates of missed canals with TLAC/LaAC using PTN—revealed that IAC and WOG combinations were more tolerant of conservative preparations, demonstrating greater robustness in challenging anatomies where unrestricted dentin sacrifice could compromise long-term tooth survival.

This conservative strategy accentuated the relative advantages of IAC, which achieved more comprehensive instrumentation than TLAC, with greater volume and surface area enlargement and substantially lower percentage of unprepared canal surface (8.0%-18.8% vs. 30.8%-36.7%). However, this enhanced instrumentation efficacy came at the cost of more aggressive removal of incisal edge and intercanal regions, resulting in significantly reduced residual “enamel” and “dentin” thickness. Given the finite element analysis findings by Galal et al. [45], which revealed significant stress concentration patterns in incisal access designs, clinicians should consider implementing adjunctive composite reinforcement strategies to compensate for structural weakening and mitigate potential fracture risks in long-term clinical applications. Furthermore, IAC improved instrument accessibility by reducing the approach angle to both buccal and lingual canals. This modification minimized coronal interference and reduced the impact of canal curvature on instrumentation, thereby decreasing stress and fatigue on endodontic instruments. Contrary to these findings, Rover et al. [22] found no significant differences in canal preparation outcomes or fracture resistance between minimally invasive cavities (IAC) and TLAC in mandibular incisors, regardless of the instrumentation system used (TRUShape 3D or MTwo). This discrepancy could potentially be explained by the inherently limited working space in minimally invasive designs, alongside differences in experimental methodology and the use of extracted teeth. Comparative data demonstrated an equally high incidence of canal straightening with LaAC (9 cases per approach), whereas no cases of canal straightening were observed in the TLAC group. Consequently, clinicians should exercise caution when employing IAC or LaAC to preserve intercanal dentin at midroot levels, particularly in teeth with pronounced proximal root concavities and distal curvature, to prevent structural compromise. In daily practice, the decision to use IAC should be followed by mandatory immediate restoration with bonded composite to reinforce the compromised incisal edge, transforming the access cavity into a strategically restored unit rather than leaving it as an inherent weak point.

The LaAC approach yielded paradoxical outcomes: although it consistently provided reliable access to lingual canals, it frequently failed to adequately instrument buccal canals when using PTN systems (100% failure in Specimen 1 replicas [8/8]; 62.5% in Specimen 2 [5/8]). This resulted in a significantly greater percentage of unprepared canal surface compared to IAC, which is a particularly noteworthy finding considering the anatomically larger dimensions characteristic of buccal versus lingual canals [22]. Substituting PTN with WOG significantly improved outcomes under LaAC, achieving complete lingual canal instrumentation and successful buccal canal preparation in 100% (8/8) and 87.5% (7/8) of Specimen 1 and 2 replicas, respectively. The unprepared surface area was significantly reduced from 47.6% to 13.9% in Specimen 1 and from 29.2% to 9.3% in Specimen 2 accordingly. This enhancement can be attributed to WOG’s gold-heated NiTi alloy, which offers superior flexibility and fatigue resistance compared to conventional instruments [33–36]. Of particular clinical relevance, both LaAC and IAC techniques were associated with canal straightening, underscoring the critical need for precise preservation of intercanal dentin to maintain root structure integrity during instrumentation. Therefore, when a LaAC is indicated (e.g., for direct lingual canal access), clinicians should preferentially select highly flexible reciprocating file systems like WOG and be prepared to accept a higher risk of canal transportation and uneven apical enlargement, as evidenced by elevated *D* and *D/d* ratio values. The imperative for complete debridement must be carefully weighed against these geometric compromises.

Despite its esthetic advantages and access efficiency, TLAC rarely permits straight-line instrumentation in anterior teeth due to misalignment between the coronal and radicular long axes [28]. Reported success rates are as low as 10% in maxillary central and 0.8% in lateral incisors, with no successful cases documented in mandibular incisors [46–48]. In double-canaled incisors, our results confirm that TLAC critically limits the clinician’s ability to locate, negotiate, and fully debride the root canal system. Liu et al. [16] observed that while conservative assess cavities preserved dentin and improved fracture resistance in mandibular premolars with Vertucci type V canals, they impeded instrumentation and obturation. In cases with severely curved lingual canals, a modified conservative access, involving partial removal of pericervical dentin to reduce angulation, yielded outcomes comparable to conventional access cavities [16]. Our study adds a nuanced perspective in mandibular incisors with Vertucci type III canals: although TLAC and LaAC better preserve incisal and intercanal structure, they frequently proved ineffective in ensuring complete debridement, often resulting in a missed buccal or lingual canal. In contrast, the more extended IAC, despite sacrificing some incisal structure, provided the most direct and predictable access to both canals (as evidenced by its minimal proportion of unprepared surface), underscoring the inherent trade-off between structural preservation and procedural efficacy in contemporary endodontic planning. These findings align with the view of Shabbir et al. [28] that MI access designs, in certain contexts, pose more risks than benefits to endodontic outcomes. For the practicing clinician, this underscores a fundamental principle: when preoperative diagnosis confirms the presence of two canals in a mandibular incisor, prioritizing a design like IAC that ensures complete debridement often outweighs the marginal benefits of ultra-conservative dentin preservation, as leaving infected tissue poses a greater threat to long-term survival than the structural compromise.

It is widely accepted that instrumentation system selection must align with root canal anatomy to optimize debridement [26]. Regardless of the system used, persistent unprepared surfaces remain a concern, as they may harbor microbes and impede irrigant penetration, contributing to post-treatment infection [34, 35, 38]. Our findings demonstrate distinct preparation patterns: while TLAC using PTN/WOG systems consistently achieved complete buccal canal preparation but failed to instrument lingual canals, LaAC with PTN successfully accessed lingual canals while inadequately preparing buccal canals. Notably, LaAC with WOG instrumentation effectively addressed these limitations and achieved near complete dual canal preparation, with only one exceptional case in Specimen 2. However, a comparative analysis revealed a substantially higher incidence of canal straightening with WOG (15/48) than with PTN (5/48), demonstrating a clinically relevant compromise between instrumentation completeness and anatomical conservation. A critical distinction between the two NiTi systems lies in their kinematics: continuous rotation (PTN) versus reciprocation (WOG). Continuous rotation can generate a pronounced “screw effect” increasing the risk of instrument engagement and torsional stress in the confined, complex anatomy of Vertucci Type III canals. This may explain PTN’s failure to adequately prepare buccal canals via LaAC. In contrast, WOG’s reciprocating motion mitigates this effect, reducing overall stress and enhancing its ability to follow severe curvatures. This combination of kinematic efficiency and enhanced alloy flexibility was pivotal in achieving more complete debridement with LaAC, albeit at the cost of a higher incidence of canal straightening, highlighting the inherent compromise between preparation efficacy and anatomical conservation. In collection, WOG’s reciprocating kinematics and alloy properties improve debridement in incisors with Vertucci Type III canals, while PTN’s limitations underscore the need for instrument selection tailored to the access design. These findings directly inform clinical instrument selection: for complex dual-canal mandibular incisors, particularly when a LaAC is employed, the use of a more flexible reciprocating system is advised. However, this approach must be coupled with meticulous glide path preparation and frequent patency checks to mitigate the risks of canal transportation and other procedural errors. 

Data in Tables 4 and 5 demonstrate a significant influence of tooth prototype, access cavity design, and instrumentation on post-preparation apical diameters. Among Specimen 1 replicas, the LaAC/WOG group exhibited the largest *D* and the highest *D/d* ratio. For Specimen 2, the LaAC/WOG combination again produced the greatest *D*, while the TLAC/WOG group recorded the most elevated *D/d* ratio. This apical enlargement pattern resulted from the WOG technique’s instrumentation of both canals within the 1-2-1 configuration, which led to dual preparation of the common apical foramen. Owing to the steep access angle and prolonged instrumentation time, particularly when preparing the buccal canal via LaAC, a pronounced bucco-lingual enlargement occurred at the curvature plane, thereby directly increasing both the *D* dimension and the *D/d* ratio.

This study further demonstrated a significant correlation between anatomical dimensions of the root canal and instrumentation outcomes. Anatomical constraints, particularly severe curvatures at bifurcation and convergence zones, posed considerable operative challenges during canal exploration and preparation. A striking finding was the exclusive occurrence of apical perforations in Specimen 2 replicas, with an incidence rate of 45.8% (22/48). Quantitative analysis revealed distinct preparation patterns between the two replica groups. The constricted canals (Specimen 2 replicas) exhibited significantly smaller absolute increases in canal volume and surface area after instrumentation. It is important to note that the difference in root canal volume between the two specimen types prior to preparation, despite the use of identical size NiTi rotary files, may have influenced these outcomes. Given their notably smaller baseline dimensions (approximately half those of the Specimen 1 replicas) and absence of complex isthmus anatomy, the constricted canals demonstrated both a greater percentage of enlargement (volume: 179.8%-355.8% vs. 75.8%-164.4%; area: 56.8%-105.9% vs. 24.6%-55.1%) and a significantly smaller proportion of unprepared surface areas (8.0%-34.2% vs. 11.8%-47.6%) compared to the spacious canals. These findings suggest that while constricted canals undergo less net dentin removal, they achieve more homogeneous preparation due to enhanced instrument-to-canal wall contact in confined spaces and their characteristically simpler, more circular cross-sectional geometry. Clinically, the markedly higher percentage of enlargement in constricted canals underscore their susceptibility to over-preparation and specific errors, including apical perforation, canal straightening, and instrument separation, even with standardized instrumentation protocols. Conversely, the higher unprepared surface area in spacious canals with isthmus anatomy emphasizes the necessity of pre‑operative canal geometry assessment, as it critically influences debridement predictability and biofilm eradication potential. These findings highlight the importance of recognizing these instrumentation patterns to guide clinical decisions, particularly in selecting appropriate access cavity designs and instrumentation strategies for mandibular incisors with two root canals. Collectively, the data establish that access design, instrument selection, and canal anatomy are critical determinants of preparation outcomes. This compelling evidence necessitates the rejection of the null hypothesis.

This study has several limitations that should be acknowledged. First, while the 3D-printed teeth were designed to represent varying degrees of operational difficulty, they were based on only two human specimens. This restricted sample size may introduce selection bias and limit the generalizability of findings to other anatomical variations. Second, the selected specimens exhibited Vertucci Type III canal configurations, where the buccal and lingual canals merged apically. This does not account for the full spectrum of possible anatomical variations, including other Vertucci classifications that may present different instrumentation challenges. Third, the use of established NiTi systems (PTN and WOG), while methodologically controlled, may limit the extrapolation of our findings to newer generation instruments with potentially superior flexibility. Additionally, instrumentation was performed at room temperature to maintain resin model integrity, though this may limit direct simulation of NiTi phase transformations at body temperature. Fourth, the standardized instrumentation protocol intentionally adopted a conservative access strategy with limited pericervical dentin removal and omitted adjunctive orifice flaring techniques. While this enhanced standardization and relevance to dentin-preserving principles, it increased technical difficulty compared to unrestricted clinical preparations and may overestimate challenges in negotiation and shaping, particularly in resin models where material properties differ from natural dentin. This could limit direct extrapolation to clinical scenarios permitting more aggressive access modification. Finally, although resin models provide controlled testing conditions, their mechanical properties, particularly hardness and elasticity, differ from natural dentin, and this may lead to overestimation of the incidence and degree of procedural errors. These material disparities could potentially influence instrumentation outcomes and should be considered when interpreting the results. While this investigation offers valuable preliminary data, further validation using natural teeth specimens and clinical studies is warranted before broader application of these findings.

## Conclusions

Access design and file selection significantly impact instrumentation efficacy in challenging Vertucci Type III mandibular incisors. The IAC demonstrated superior performance over TLAC and LaAC approaches, while WOG outperformed PTN, particularly in enhancing LaAC outcomes. These findings highlight the value of IAC and WOG combinations for achieving more robust instrumentation under conservative conditions that prioritize dentin preservation.

## Data Availability

The data that support the findings of this study are available from the corresponding author upon reasonable request.

## Abbreviations

3D: Three-dimensional
ANOVA: One-way analysis of variance
IAC: Incisal access cavity
CBCT: Cone-beam computed tomography
CEC: Conservative endodontic cavity
CEJ: Cement enamel junction
DNS: Dynamic navigation system
LaAC: Labial access cavity
LSD: Least significant difference
micro-CT: Micro-computed tomography
MI: Minimally invasive
NiTi: Nickel-titanium
TLAC: Traditional lingual access cavity
WL: Working length
WOG: WaveOne Gold
PTN: ProTaper Next
STL: Standard triangle language
